# Osseodensification technique in crestal maxillary sinus elevation—A narrative review

**DOI:** 10.1111/cid.13399

**Published:** 2024-10-01

**Authors:** João Gaspar, Ziv Mazor, Estevam A. Bonfante

**Affiliations:** ^1^ Department of Oral Surgery, Egas Moniz Center for Interdisciplinary Research Egas Moniz School of Health and Science Caparica Portugal; ^2^ Private Practice Tel Aviv Israel; ^3^ Department of Prosthodontics and Periodontology University of São Paulo – Bauru School of Dentistry Bauru SP Brazil

**Keywords:** dental implants, membrane perforation, Osseodensification, Osseointegration, sinus floor elevation

## Abstract

Osseodensification is a novel approach that has significantly advanced the field of implant dentistry, particularly in the context of transcrestal maxillary sinus floor elevation. This technique involves the use of specially designed burs that compact and densify bone along the osteotomy walls, thereby enhancing implant primary stability and facilitating osseointegration in low‐density bone. This article reviews the historical evolution of implant site preparation, and the biomechanical, histological, and clinical evidence of osseodensification with a special focus on its application in sinus floor augmentation. The integration of this technique into contemporary practice represents a paradigm shift, offering a minimally invasive and efficient solution for addressing the challenges of posterior maxilla, with improved patient‐reported outcomes and low complication rate. Three different protocols for sinus lift and implant placement using osseodensification burs are proposed based on available literature, and risk factors for Schneiderian membrane perforation based on residual bone height are discussed, along with implant‐related outcomes and patient‐reported outcome measures. The potential for osseodensification to become a standard practice in sinus floor augmentation is emphasized, highlighting key aspects such as surgical protocol and patient selection.

**Summary box**

**What is known**

- Maxillary atrophy presents several reconstructive challenges for implant dentistry and further discussion of available techniques is necessary.
- Atemporal implant stability has been shown in low‐density bone with osseodensification drilling and benefits for sinus floor elevation have been presented.

**What this study adds**

- Protocols for transcrestal sinus floor elevation using osseodensification drilling are presented for areas with residual bone height of ≥2 and ≤6 mm.
- Residual bone height of ≤2–3 mm is suggested as a risk factor for sinus membrane perforations using osseodensification burs.

## 1 INTRODUCTION

Modern dental implantology has paved the way as a predictable treatment option for edentulous patients eventually becoming a standard of care. Because high survival rates may be achieved in the long‐term from single‐unit to full‐arch reconstructions, many patients continuously benefit from this treatment modality with significant improvement in their quality of life.^1^ The field continues to evolve rapidly with significant attention devoted to the development of new implant surfaces, subtractive and additive manufacturing of new restorative materials, the use of digital workflows that expedite treatment time, and the treatment of diseases and implant‐related complications.^2^

Different from new implant designs, surgical drilling and instrumentation techniques for implant site preparation evolved at a much slower pace. In the 1980s, the main concerns to achieve and maintain successful osseointegration were (1) to avoid bone overheating during the drilling procedure. Thus, to avoid bone necrosis due to heating above temperature thresholds (between 47 and 55°C for 1 min),^3^ conventional implant site preparation techniques have advocated progressive drilling using successively increasing diameter clockwise twisted drills that subtracted bone when rotated from 800 to 1200 rpm under copious irrigation^4^ (2) To obtain sufficient implant primary stability, which was generically perceived by peak implant insertion torque values (IT); and (3) to ensure implants were allowed to heal without any load for a period of 3–6 months.^5^, ^6^, ^7^, ^8^, ^9^

Early implant treatments were mainly focused in the restoration of edentulous arches, and it was only later that publications documented high survival rates for single unit and partially edentulous cases.^10^, ^11^ The advances in microroughened implant surfaces fostered early prosthetic loading protocols, then reduced to 6–8 weeks, and subsequently to immediate loading protocols initially in full‐arch reconstructions.^12^, ^13^, ^14^ Clearly, such encouraging results eventually lead to the development of the immediate implant placement concept,^15^, ^16^, ^17^ which currently has defined criteria and evidence‐based protocols for single implants placed in the anterior or posterior maxilla and mandible.^18^

Considering that goals of implant treatment include not only the reestablishment of function, esthetics, and long‐term stability but also the improvement of patient‐reported outcomes including reduction in treatment times, surgical interventions, and low morbidity.^19^ The aim of this review is to discuss advances in surgical drilling techniques targeted to benefit patient implant‐supported rehabilitations. Comparative evaluations of implant site preparation techniques attempting to improve implant stability will be presented, with special attention to challenging scenarios of the resorbed posterior maxilla demanding sinus elevation procedures for successful implant placement. Protocols for treatment in these scenarios using osseodensification burs will be discussed. The background and rationale for this procedure will be provided below. The key questions to be addressed are how can osseodensification burs used for sinus floor elevation be compared to traditional techniques in terms of marginal bone levels, implant survival rate, patient‐reported outcome measures, and involved risk factors. Eligible studies came from literature published in English language, with no limitation for search year, animal and mainly clinical studies, with searches performed in Pubmed/MEDLINE, Scopus, and Google Scholar.

## 2 IMPLANT STABILITY, SURGICAL DRILLING, AND BONE QUALITY

Implant stability has been an accepted criteria for immediate loading, where several studies include varied values of IT to define the presence or absence of implant stability.^18^ Actually, the values of IT are in many publications the main parameter used for immediate loading and they may range from ≥15 to ≥50 N.cm.^20^ Implant stability has been defined as a relative mobility of an implant in relation to its surrounding bone when tested manually or with a motion‐sensing device.^21^ Implant stability histological development has been comprehensively described where an initial mechanical stability (primary stability) takes place due to compressive contact and friction between the implant surface and the prepared osteotomy walls. Then a series of healing events, including necrosis and subsequent resorption of traumatized bone around the implant body, occur until new bone formation and biologic stability (secondary stability) is achieved to allow prosthetic loading.^22^ Some factors that affect primary implant stability include bone quantity and quality, the surgical drilling technique, and implant features from its macrodesign to surface treatment.^23^ Primary stability seems to be the basis for the determination of the prosthetic loading protocol.^24^ The factors that are implicated in secondary implant stability are primary stability, bone remodeling, and implant design features.^25^, ^26^

Because alveolar bone density and cortical bone thickness is significantly associated with primary implant stability, different treatment planning and surgical drilling procedures are needed in areas of lower bone density to improve primary stability and osseointegration.^27^ In the presence of micromovement due to poor primary implant stability, a fibrous tissue capsule can be formed around the implants eventually leading to osseointegration failure.^28^ Also, low levels of primary implant stability may hinder bone remodeling after implant placement potentially leading to failure of the device.^29^

Several measurement tools have been used for the quantitative evaluation of primary implant stability including peak insertion torque values (IT), implant stability quotient (ISQ), and the periotest value (PTV). Insertion torque values can quantitatively measure the torque required to place the implant in the prepared osteotomy site using manual or electronic drill devices and it is expressed in Newton centimeter (N.cm). In brief, it measures the frictional resistance to rotation which is uniquely dependent on the interplay between implant design, surgical drilling technique, and bone quality.^30^ The implant thread design affects the cutting torque and may challenge the theoretical correlation between IT and implant micromovement.^31^ Also, IT values may be useful only in the assessment of primary implant mechanical stability since secondary stability clinical assessment cannot be made using a torque wrench without compromising osseointegration.^32^, ^33^ The implant stability quotient (ISQ) has been introduced and defined as a parameter that is related to time, and it is the ratio used to evaluate implant and/or abutment stability using resonance frequency analysis (RFA).^21^ The RFA is a noninvasive method that has been widely used, and it comprises the determination of the relative stiffness of an implant within the bone via attachment of a resonance frequency transducer containing two piezo‐ceramic elements to an implant. One piezo element is excited by an electrical signal, and the resulting vibration stimulus comprised of small sinusoidal signals (range of 5–15 kHz) is measured by the second element. The peak amplitude of the response is then encoded into the ISQ parameter which ranges from 0 to 100 where the higher the resulting frequency, expressed in kHz, the stiffer the implant‐to‐bone connection.^34^ Finally, the periotest value involves the percussion of the implant using the periotest device and it ranges from −8 to +50, where the lowest values reflect higher implant stability.^35^

A conceptual graph of implant stability temporal development has suggested that adequate primary stability plays a dominant role for implant secondary stability during the first week after implantation, and thereafter it decreases significantly to minimal levels between weeks 2 and 3, only recovering due to new bone formation (secondary stability) at about 5 weeks.^36^ Although there is commonly a correlation between high IT and primary stability, there is no correlation between high implant primary stability achieved through high insertion torques (>50 N.cm) due to underdrilling and subsequently secondary stability.^37^ However, there appears to be a correlation between IT and ISQ values.^38^ The clinical usefulness of ISQ measurement as a substitute parameter for IT to measure the implant primary stability remains and will always be controversial,^39^ since surgical drilling techniques, bone quality, and implant designs vary substantially among studies.^40^

A commonly used surgical approach to reach higher primary implant stability, mainly in low‐density bone, comprises the implant placement in a substantially smaller osteotomy. This technique, known as undersized or under‐preparation drilling, is usually achieved by skipping the use of the last drill from the manufacturer recommended conventional drilling protocol eventually resulting in a substantial increase in IT values. In the underdrilling technique, there is a trade‐off between gain in primary implant mechanical stability and the resulting loss in secondary stability due to microcrack formation and compression bone necrosis that trigger bone extensive remodeling.^26^, ^41^, ^42^, ^43^, ^44^, ^45^ However, an in vivo study has shown that the rabbit bone tissue can accept certain levels of compressive strain caused by oversized implants designed with predetermined static strain as well as beyond ultimate strain, without compromising the osseointegration process observed at 24 days.^43^

A systematic review with meta‐regression analysis evaluating the up to 15 months noninfectious marginal bone level changes of conventional and underprepared drilling osteotomy sites based on bone density has shown a significant correlation between underdrilling and marginal bone loss in bones D1, D2, and D3.^46^ Moreover, when inserted with significantly high IT, the implant‐abutment connection may be deformed compromising the prosthetic reconstruction,^47^ as well as alter the surface roughness properties of implants.^45^, ^48^ Low insertion torque (<30 N.cm) has been reported to increase the possibility of implant early failure by 14× in comparison to insertion torque of higher than 30 N.cm.^49^ On the other hand, IT values ≥80 N.cm did not prevent osseointegration or increased bone resorption in early loading senarios.^50^

## 3 OSSEODENSIFICATION DRILLING (OD)

With the aim of creating an implant preparation site avoiding subtractive bone drilling, a novel biomechanical bone instrumentation method was created by Huwais and coined as osseodensification.^51^ Specially designed burs (Densah® bur, Versah, Jackson, Michigan, USA, hereon OD bur or densifying bur) have been tailored to be used in high speed with a slow, incremental process to preserve collagen and to enhance bone plasticity and reproduce osseodensification. Osseodensification burs have lands with a large negative rake angle that work without cutting to densify trabecular bone by compacting and autografting bone into the trabecular space.^52^ Since collagen provides trabecular bone its toughness and its ability to dissipate energy,^53^ conventional bone subtractive drilling will deteriorate collagen integrity, which has been found to be directly linked to bone plasticity.^54^ The bone plastic deformation occurs as a gradual change, which is dependent on time and strain rate.^55^, ^56^ Bone fluid content also plays an important role in determining bone viscoelasticity.^57^ It is a time‐dependent process,^52^, ^57^ thus in order to achieve bone plasticity and enhance bone toughness, it is paramount to rethink bone instrumentation, specifically through subtractive drilling and consider a method to apply time dependent strain in a controlled manner.

The densifying burs have been designed with a chisel edge and a tapered shank that progressively increases diameter, controlling the expansion process. They can be used in the standard surgical motor with irrigation and are dual action, where counterclockwise (CCW) rotation at 800–1200 rpm results in noncutting and bone densification, whereas clockwise (CW) rotation in the same rpm range produces bone cutting. In contrast to conventional drilling recommended by dental implant manufacturers in their proprietary surgical kits that subtract bone, the OD technique has been based on the bone elastic and plastic properties which facilitate bone bulk preservation and compaction, resulting in the autografting of osseous material into the trabecular space and enhancing its density while preserving the recommended final osteotomy dimension. Gentle compressive forces against the implant are thereby generated due to a residual strain and bone spring‐back effect that enhances primary implant stability.^52^, ^58^ An in vivo study in porcine model evaluated osseointegration parameters after alveolar ridge expansion technique in the atrophic mandible followed either by the use of manual osteotomes or by OD burs.^59^ Although, there were no differences between the evaluated techniques for mean ridge expansion dimension (80% in OD and 63% in osteotomes), and for bone‐area fraction occupancy (BAFO, 56% for OD and 31% using osteotomes), the parameters insertion torque and bone‐to‐implant contact (BIC) values were significantly higher in OD burs compared to conventional osteotomes.^59^ When surgical instrumentation was evaluated in the setting of a posterior lumbar spine fixation, improved mechanical stability was observed at 6 weeks for OD instrumentation but took longer time with conventional instrumentation (hand reaming) at 12 weeks. In this scenario, BAFO was not different between both surgical techniques, likely due to the uniformity of the bone anatomy, osteosynthesis features, and biomechanical conditions, according to the authors.^60^

With osseodensification, and due to the compaction autografting that generates the spring‐back effect, the osteotomy does not need to be undersized. Rather, it is very close to the implant major diameter (0.5 mm smaller in the maxilla and 0.2 mm smaller in the mandible); therefore, rather than the implant creates static compression of bone with standard under‐sizing drilling to gain primary stability, osseodensification produces an oversized osteotomy that is very close to the implant major diameter, so the bone would gently reverse‐compress the implant to produce the needed increased primary stability.^52^ It is also documented that the compacted autografting of bony particles into the trabecular walls fosters faster bone formation at the initial weeks by bone chip residues present between osteotomy walls and the implant.^61^ A clinical study has shown a significant increase in bone density for OD drilling compared to conventional drilling in the posterior maxilla with no detrimental effect on parameters such as marginal bone loss or osseointegration.^62^ This increase in bone density due to the compaction autografting has been maintained beyond the instrumentation day to 1 year after restoration, suggesting that implants placed in osteotomies prepared by the osseodensification burs resulted in sustained higher bone density compared to conventional drilling.^63^ The resulting increase in implant primary stability has been clinically documented to enhance immediate implant placement and healing in both maxilla and mandible with osseodensification.^64^, ^65^

Several in vivo studies have been performed to provide the biomechanical and histologic basis of osseodensification drilling (OD) in meaningful animal models simulating low‐density bone (sheep ilium).^66^, ^67^ Comprehensive comparative evaluation of manufacturer's recommended drilling protocol (regular drilling) to place either conical or parallel walled dental implants and osseodensification (OD) showed that OD resulted in significantly higher IT levels for both implant designs.^61^ Histological findings at 6 weeks showed no osseointegration impairment in OD drilled sites compared to the control subtractive regular drilling. A remarkable finding of this study was that only in OD groups bone chips, resulting from the OD bone autografting, enabled larger degrees of bone apposition toward the implant surface since they bridged the gaps between the implant and bone.^61^ A subsequent study in the same large animal model confirmed that the use of OD burs significantly increased IT levels in low‐density bone compared to regular drilling for the placement of as‐machined or acid‐etched surfaces, with no differences between them at 3 weeks. At 12 weeks, new bone formation was observed in all groups with no detrimental effect of OD protocol compared to regular drilling.^68^ In essence, along with higher IT levels when compared to regular drilling protocols, dental implants placed via OD have demonstrated atemporal biomechanical stability and osseointegration.^69^

A multicenter controlled clinical trial has shown that implants placed with OD demonstrated significantly higher IT values and sustainable secondary stability measured by ISQ at 3 and 6 weeks when compared to regular drilling protocols applied to several implant designs, regardless of location (the anterior or posterior region of the maxilla or in the posterior region of the mandible).^70^ Promising findings had been previously confirmed in multicenter clinical retrospective clinical study of a 5‐year follow‐up of 253 implants with five different implant geometries.^71^, ^72^ A double‐blind randomized clinical trial showed that in low‐density bone, implants with healing chambers presented significantly higher IT levels when placed after OD compared to undersized drilling.^73^

The use of OD drilling protocol was not limited to assure clinicians and patients of more predictable higher IT levels and maintenance of implant secondary stability without any compromise or negative effect in bone vascularity. According to a recent clinical study that compared the onset of vascular bleeding and the osteotomy blood fill between OD and conventional drilling, OD did not seem to negatively affect or induce loss of bone vascularity.^74^ Its benefit in various clinical scenarios was documented, including immediate implant placement and loading scenarios.^65^ Its use has also been shown to be beneficial for alveolar ridge expansion,^59^, ^75^, ^76^, ^77^ and for achieving bone septum expansion and high implant stability in wide sockets such as those observed in immediate molar replacements.^78^ A retrospective multicenter study with up to 5‐year follow‐up of 131 patients who received 145 immediate implants in molar extraction sockets showed a 93.1% cumulative survival rate with the use of the osseodensification technique.^64^ Since a significant increase and maintenance of higher bone density for a period of 1 year has been shown when OD drilling was used relative to regular drilling,^63^ its use for crestal maxillary sinus elevation has been documented and will be explored in the next section.

## 4 MAXILLARY SINUS FLOOR ELEVATION (SFE)

Traditionally, the posterior maxilla has been associated with a higher rate of implant failure.^79^, ^80^, ^81^, ^82^, ^83^ After tooth loss, significant resorption of the alveolar bone may occur in combination with pneumatization of the maxillary sinus, which results in limited residual bone height (RBH) below the sinus floor. Additionally, the typically low bone density in this region adds further complexity to achieve successful implant placement with proper primary stability.^80^, ^82^, ^84^

Maxillary sinus floor elevation (SFE) has emerged as a critical procedure for increasing the vertical bone dimension to facilitate implant placement.^85^, ^86^ The primary techniques for SFE include the lateral window (LW) technique and the transcrestal approach, each with its own set of advantages and technical considerations.^87^, ^88^, ^89^ The classical LW approach was initially introduced in the 1970s by Tatum^90^ and more comprehensively detailed by Boyne and James in 1980,^91^ and has still been considered the gold‐standard method when RBH is <5 mm.^92^, ^93^ Despite its predictability and effectiveness, this technique involves elevating an extensive surgical flap with at least one vertical releasing incision and therefore is associated with higher morbidity, longer recovery periods, and greater post‐operative discomfort compared to more conservative techniques.^94^, ^95^, ^96^ In contrast, a less invasive alternative for SFE by crestal approach was initially suggested in 1976 and later refined by Summers in 1994 using tapered osteotomes to break the maxillary sinus floor and lift the Schneiderian membrane.^97^ This method is traditionally recommended for patients with at least 5 mm of RBH.^93^, ^98^ According to a systematic review based on 3131 implants, implant survival rate after osteotome‐mediated SFE increased to 96.9% when RBH ≥5 mm, in contrast with 92.7% when RBH <5 mm.^99^ However, this traditional crestal sinus grafting technique is not indicated in cases with a sloped sinus floor due to the high risk of membrane perforation.^100^ In addition, the uncontrolled tapping required to fracture the sinus floor could potentially trigger benign paroxysmal positional vertigo (BPPV), causing significant distress to patients.^101^, ^102^ Despite these limitations and the fact that it is performed blindly, the transcrestal approach offers numerous benefits over the LW method: it is less invasive and time‐consuming, preserves the buccal bone wall potentially enhancing healing speed, and is associated with less risk of infection and postoperative morbidity.^96^, ^99^, ^103^ Consequently, various transcrestal techniques have been developed to mitigate the shortcomings of the original method proposed by Summers.^104^, ^105^

### 4.1 Osseodensification drilling for transcrestal sinus floor elevation

OD has emerged as a predictable and reproducible technique for facilitating transcrestal SFE.^95^, ^104^, ^106^, ^107^ Since it compacts and autografts bone along the osteotomy walls and its apex rather than removing it, this approach not only enables sinus lift via the crestal preparation but also allows to improve the overall bone density, which is particularly beneficial in the posterior maxilla where bone quality is often compromised.^52^, ^66^, ^108^ The recommended technique for transcrestal sinus augmentation through OD involves utilizing the OD burs in CCW (osseodensifying mode), in a pumping action with copious irrigation.^104^ In addition to prevent overheating, this combination of the fluid pumping motion and high‐speed CCW rotation generates a hydrodynamic wave ahead of the tip of the burs, known as a compression wave. Once the densifying bur penetrates the sinus floor, the irrigation solution and the bone debris hydraulically lift the Schneiderian membrane.^52^, ^104^

Sinus graft via osseodensification drilling differs significantly from the reamer approach. The reamer approach is a subtractive instrumentation method that facilitates breaching the sinus floor to reach the sinus membrane with lower risk of tearing it, but it will not facilitate lifting the membrane. Therefore, further second method/tool is needed to lift the membrane off the bony bed, and a third method/tool to graft bone or biomaterials into the lifted space. Osseodensification facilitates the above three steps with one instrument, that safely breaches the sinus floor, lifting the membrane off the body bed and simultaneously compacting autogenous bone from the osteotomy walls into the lifted space up to 3 mm. Additional bone grafting can be achieved using the same osseodensification burs to graft biomaterials to secure additional lift volume.^104^

#### 4.1.1 Clinical evidence

Several clinical studies have demonstrated the effectiveness and predictability of OD for transcrestal SFE in clinical scenarios with RBH as low as 2 mm with a vertical increase in postgrafting of up to 10 mm.^95^, ^104^, ^106^, ^107^ However, similar implant survival rates with OD transcrestal SFE were reported by other authors in cases with severe posterior maxillary atrophy with RBH as low as 2 mm,^95^, ^109^ without the drawbacks inherent in both LW and Summers' techniques. OD sinus grafting protocols have demonstrated their effectiveness to elevate the sinus floor and simultaneously improve implant stability.^109^

Saglanmak et al.^107^ also conducted a retrospective study to radiographically assess the endo‐sinus bone gain following OD SFE. Patients were divided into two groups: OD group, in which SFE was achieved exclusively by using densifying burs as will be subsequently described in Protocol I; and ODA—osseodensified augmentation group, in which SFE was performed with additional propelling of grafting material into the sinus as described in Protocol II. The authors concluded that SFE with OD technique seems to be a fast, effective, and safe method, either with or without application of grafting material.

**FIGURE 1:**
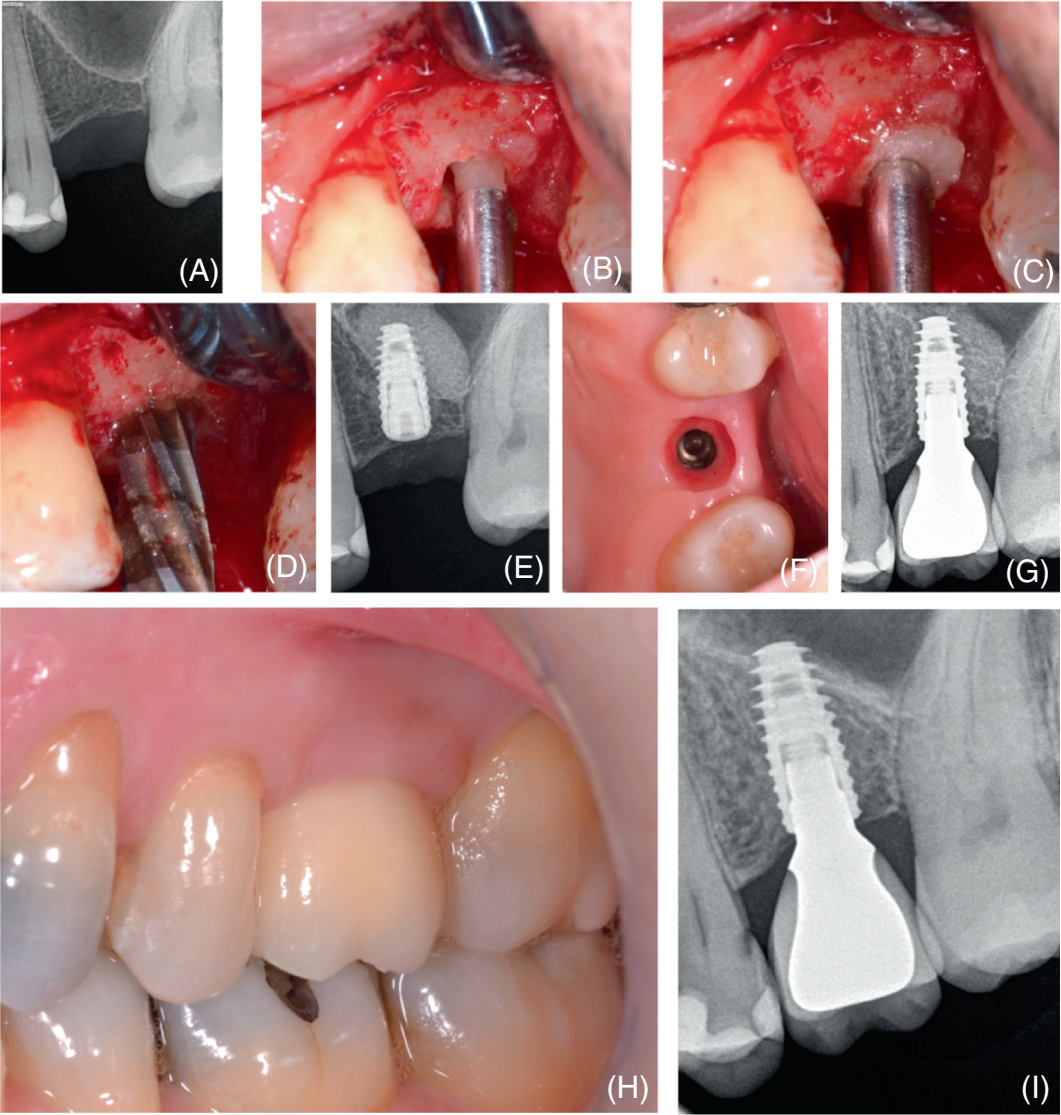
Sinus floor elevation with osseodensification in a case with an oblique sinus floor: (A) baseline periapical radiograph; (B,C) application of grafting material post‐membrane lift via use of OD bur; (D) propelling of graft material with OD bur; (E) post‐operative radiograph; (F) peri‐implant tissue health before final impression; (G) follow‐up at 6 months after final crown insertion; (H,I) 4‐year follow‐up.

As previously discussed, a frequent challenge with the transcrestal approach occurs in areas where the maxillary sinus floor is slanting. A recent prospective clinical study^110^ with 16 patients aimed to assess the efficacy of SFE using OD technique in cases with a RBH of 4–7 mm and an oblique sinus floor (Figure 1), where the traditional Summers technique is associated with a high rate of membrane perforation.^101^, ^111^ The authors demonstrated that OD was an effective and safe procedure, without an increased risk of Schneiderian membrane perforation in sites with moderate RBH and a sloping sinus floor. The sinus membrane integrity was assessed by direct clinical examination and a post‐operative cone beam computed tomography scan. Only one membrane perforation was reported, with a 100% implant survival rate at the 1‐year follow‐up.^110^

Regarding peri‐implant bone level, a split‐mouth study where the same patient received implants in the posterior maxilla placed either after conventional subtractive osteotomy or by using osseodensification drilling showed no statistical difference between the levels of crestal bone when evaluated up to 6 months.^112^ Implant survival of 100% has been reported when OD was used for transcrestal sinus floor elevation in prospective studies at 6 months^109^ as well as at 12 months.^113^ In a randomized controlled trial, comparing osseodensification drilling with lateral window technique for sinus floor elevation both techniques resulted in early implant success rate of 100% and all implants were successfully restored with screw‐retained zirconia crowns.^95^ A large retrospective clinical study showed a cumulative implant survival rate of 97% in an up to 5‐year follow‐up with sub‐sinus residual bone height of 2–7 mm with an average of 5.4 mm at baseline resulting in a vertical augmentation gain of 3–11 mm.^104^

#### 4.1.2 Patient‐reported outcome measures

A recent randomized clinical trial^95^ concluded that OD was as effective as LW in SFE with simultaneous implant placement when RBH ≤4 mm, but with significantly improved outcomes in terms of pain experience, impact on self‐perceived quality of life, surgery duration, postoperative edema, and analgesics intake. Postoperative symptoms self‐reported by patients as edema, hematoma, and epistaxis were all significantly more prevalent in the LW group. Farina et al.^114^ also observed a significantly lower incidence of swelling, bruising, and nasal bleeding with transcrestal SFE compared to the classical LW approach. However, the surgical act of tapping the osteotome should not be underestimated since it may cause more post‐operative discomfort during the first days of healing.^100^

#### 4.1.3 Schneiderian membrane perforation rate associated with OD

Sinus membrane perforation is the most common intraoperative complication reported in the literature during SFE with a rate of 7%–58%.^96^, ^101^, ^115^ As a consequence, it may lead to additional complications such as graft displacement into the sinus or infection. Maxillary sinus membrane perforation diagnostic methods differ and still lack proper validity. Although the comparison between the lateral window and crestal approach perforation rate is commonly made, it can be biased and therefore the results should be interpreted with caution.^116^ However, the main outcome of interest, which is membrane perforation, still needs to be examined and evaluated according to each method of diagnostic measures.

A multicenter clinical study was conducted to evaluate the membrane perforation rate during transcrestal SFE using OD in molar and premolar areas with RBH ≥2 and ≤7 mm, and also to identify the risk factors associated with this outcome. Most SFE procedures were performed in the molar region in healed bone sites and the average RBH was 5.1 mm. Sinus membrane perforation rate was low, occurring in only 7.31% (49 sites) out of 670 sites,^117^ in contrast with other clinical studies that reported a higher prevalence of perforation with LW and Summers' osteotome technique.^111^, ^118^, ^119^ Residual bone height ≤3 mm presented a risk factor for sinus membrane perforations using OD burs.^117^ In this study,^117^ high magnification with surgical suction were used to diagnose any perforation, and regardless of size, a perforation was reported as a perforation. If perforation was detected, it was registered, and grafting was not done as well as implant placement. If perforation was not detected using extensive lighting and magnification, grafting and implant placement were done, then post placement radiographs were taken to second rule out any possible missed perforation. In addition, common sequelae following sinus membrane perforations, such as sinusitis, epistaxis, oroantral communication, nasal cavity penetration, exfoliation of graft particles from the nose, and maxillary ostium obstruction,^120^ were not observed in the reported study, which reassured that clinically detectable membrane perforations were duly accounted for. While the Valsalva maneuver is the most commonly reported method used to evaluate Scheneidarian membrane perforation, its diagnostic accuracy is subject to debate, and it may lead to underestimated diagnostic of perforation rates as a result of possible false negatives.^114^

In a recent randomized clinical trial that compared SFE by crestal approach with OD versus LW technique, there were significantly more Schneiderian membrane perforations in the LW group (*p* < 0.001).^95^ In another prospective clinical study with 20 crestal OD SFE, no membrane perforations were observed, which was confirmed by cone‐beam CT scan postoperatively.^109^

### 4.2 Osseodensification crestal sinus lift protocols

The amount of RBH present in sinus floor elevation procedures has led to the documentation of different approaches using OD drilling, which will be presented in this section. It is important to note that outcome comparisons of OD drilling with lateral window techniques for sinus lift elevation are mostly suited for Protocols II and III, since the LW is mainly indicated when residual bone height is <5 mm.^92^, ^93^, ^121^ The suggested OD drilling protocols, along with cited literature, are aimed to obtain the most of the use of OD bur procedure benefits while reducing risks of sinus membrane perforation, especially in severe atrophy cases. Because the included literature cited within the suggested protocols involved the use of only one OD bur system (Densah® bur, Versah, USA), it will simply be referred to as OD bur or as densifying bur in this section. Future RCTs are encouraged to further support the protocols presented below.

#### 4.2.1 Protocol I: minimum RBH ≥6 mm, minimum alveolar width needed = 4 mm

Protocol I refers to cases where minimum RBH is equal to or more than 6 mm and minimum alveolar width is ~4 mm. A randomized controlled trial has compared OD drilling to osteotome for sinus lifting with simultaneous implant placement, without grafting (RBH = 5–8 mm). Implant success rates were 100% after 6 months, and although bone height gain was not significantly different between techniques (3.2 mm), OD drilling resulted in significantly higher bone density.^122^ Another RCT comparing OD drilling to osteotome in cases of RBH of 8 mm reported similar radiographic outcomes for both techniques, although the vertigo rates were 100% for the osteotome‐treated patients.^123^ A prospective study comparing OD drilling to osteotome for SFE and implant placement in cases of RBH of at least 5 mm showed that both immediately and at 6 months, significantly higher primary implant stability as well as bone gain was reported for OD drilling relative to osteotome technique.^124^ The last two RCTs have been included in a recent systematic review and meta‐analysis that evaluated implant stability and increase in bone height in SFE using OD drilling and the osteotome technique, and shown a moderate risk of bias.^125^

#### 4.2.2 Clinically suggested steps with OD Sinus Protocol I, minimum alveolar width needed = 4 mm

Step 1: measure bone height to the sinus floor.

Flap the soft tissue using instruments and technique normally used.

Step 2: pilot drill to 1 mm below the sinus floor.

In cases where posterior residual alveolar ridge height is ≥6.0 mm, and additional vertical depth is desired, drill to the depth determined within an approximate safety zone of 1.0 mm from the sinus floor using a pilot drill (clockwise drill speed 800–1200 rpm with copious irrigation). Confirm pilot drill position with a radiograph.

Step 3: OD bur (2.0) OD mode to sinus floor.

Depending upon the implant type and diameter selected for the site, begin with the narrowest densifying bur VT1525 (2.0). Change the surgical motor to reverse‐densifying mode (counterclockwise drill speed 800–1200 rpm with copious irrigation). Begin running the bur into the osteotomy. When feeling the haptic feedback of the densifying bur reaching the dense sinus floor, stop and confirm the first OD drilling bur vertical position with a radiograph.

Step 4: enter with OD bur (3.0) OD mode up to 3 mm past the sinus floor.

Use the next wider densifying bur VT2535 (3.0) in densifying‐mode (counterclockwise drill speed 800–1200 rpm with copious irrigation) and advance it into the previously created osteotomy with modulating pressure and a pumping motion. When feeling the haptic feedback of the bur reaching the dense sinus floor, modulate pressure with a gentle pumping motion to advance past the sinus floor in 1 mm increments. Maximum possible advancement past the sinus floor at any stage must not exceed 3 mm. Wider diameter densifying burs are used according to planned implant diameter, with the same modulating pressure and the gentle pumping motion to advance past the sinus floor in 1 mm increments with maximum possible advancement past the sinus floor at any stage not to exceed 3 mm. As the next wider OD bur advances in the osteotomy, additional autogenous bone will be pushed toward the apical end to achieve additional vertical depth, and a maximum membrane lift of 3.0 mm. Confirm the bur vertical position with a radiograph.

**FIGURE 2:**
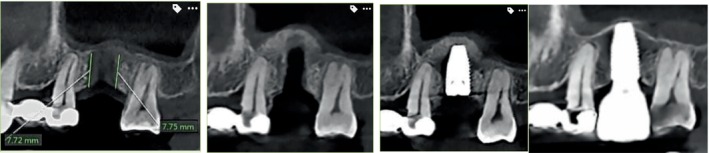
Sinus grafting with osseodensification Protocol I; with ≥7 mm to the sinus floor with immediate placement of a 5/11 mm implant with 4 years of follow‐up. Clinical case is a courtesy of Dr.Chang WenPing.

**FIGURE 3:**
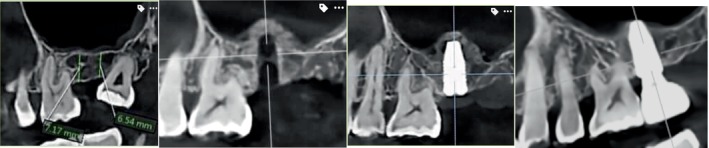
Sinus grafting with osseodensification Protocol I; ≥ 6–7 mm to the sinus floor with immediate placement of a 5/11 mm implant with 3 years of follow‐up. Clinical case is a courtesy of Dr. Chang WenPing.

Step 5: implant placement. Place the implant into the osteotomy. If using the surgical motor to tap the implant into place, the unit may stop when reaching the placement torque maximum. Finish placing the implant to depth with a torque indication ratchet wrench. Clinical cases of protocol I are illustrated in Figures 2 and 3.

#### 4.2.3 Protocol II: minimum RBH 4–5 mm, minimum alveolar width needed = 5 mm

When the minimum RBH ranges from 4 to 5 mm, minimum alveolar width needed is of 5 mm, a different surgical OD drilling approach is suggested, as presented below. The previously detailed retrospective multicentered study covered both protocols I and II for SFE and reported a 97% implant survival rate after an up to 5‐year follow‐up.^104^ RCT trials, evaluated in a systematic review and meta‐analysis as low risk of bias, compared OD drilling with osteotomes for protocol II.^125^ The amount of bone gain was similar for OD drilling compared to conventional osteotomes, but pain perception was reduced after surgery in OD drilling.^126^ Another RCT reported higher mean primary and secondary stability for OD drilling compared to osteotome, with both presenting as successful techniques for SFE.^127^

#### 4.2.4 Clinical suggested steps with OD sinus Protocol II

Step 1: measure bone height to the sinus floor.

Flap the soft tissue using instruments and technique normally used.

Step 2: OD bur (2.0), OD mode to sinus floor.

Avoid using a pilot drill. Depending upon the implant type and diameter selected for the site, begin with the narrowest densifying bur VT1525 (2.0). Change the surgical motor to reverse‐densifying mode (counterclockwise drill speed 800–1200 rpm with copious irrigation). Begin running the bur into the osteotomy until reaching the dense sinus floor. Confirm bur position with a radiograph.

Step 3: enter with OD bur (3.0), OD mode up to 3 mm past the sinus floor.

Use the next wider densifying bur VT2535 (3.0) and advance it into the previously created osteotomy with modulating pressure and a pumping motion. When feeling the haptic feedback of the bur reaching the dense sinus floor, modulate pressure with a pumping motion to advance past the sinus floor in 1 mm increments, up to 3 mm. Maximum bur advancement past the sinus floor, at any stage, must not exceed 3 mm. Bone will be pushed toward the apical end and will begin to gently lift the membrane and autograft compacted bone up to 3 mm. Confirm the bur vertical position with a radiograph.

Step 4: OD bur VT3545 (4.0), VT4555(5.0) in OD mode up to 3 mm past the sinus floor. Use the sequential wider densifying burs in densifying mode, according to planned implant diameter, in densifying mode (counterclockwise drill speed 800–1200 rpm) with copious irrigation with pumping motion to achieve additional osteotomy width with maximum membrane lift of 3 mm (in 1 mm increments) to reach final desired width for implant placement. OD burs must not advance more than 3 mm past the sinus floor at all times regardless of the OD bur diameter.

Step 5: propel allograft/bone substitute.

After achieving the final planned osteotomy diameter, fill the osteotomy with a well hydrated, mainly cancellous, allograft, or alloplast putty. Use the last OD bur used in step 4 in densifying mode (counterclockwise) with low speed 150–200 rpm with no irrigation to propel the well‐hydrated allograft/ alloplast putty into the sinus. The OD bur must only facilitate the allograft material compaction to further lift the sinus membrane, and not to advance beyond the sinus floor more than 2–3 mm. Repeat the graft propelling step to facilitate additional membrane lift as needed according to implant length.

Step 6: place implant.

Place the implant into the osteotomy. If using the surgical motor to tap the implant into place, the unit may stop when reaching the placement torque maximum. Finish placing the implant to depth with a torque indicating wrench.

**FIGURE 4:**
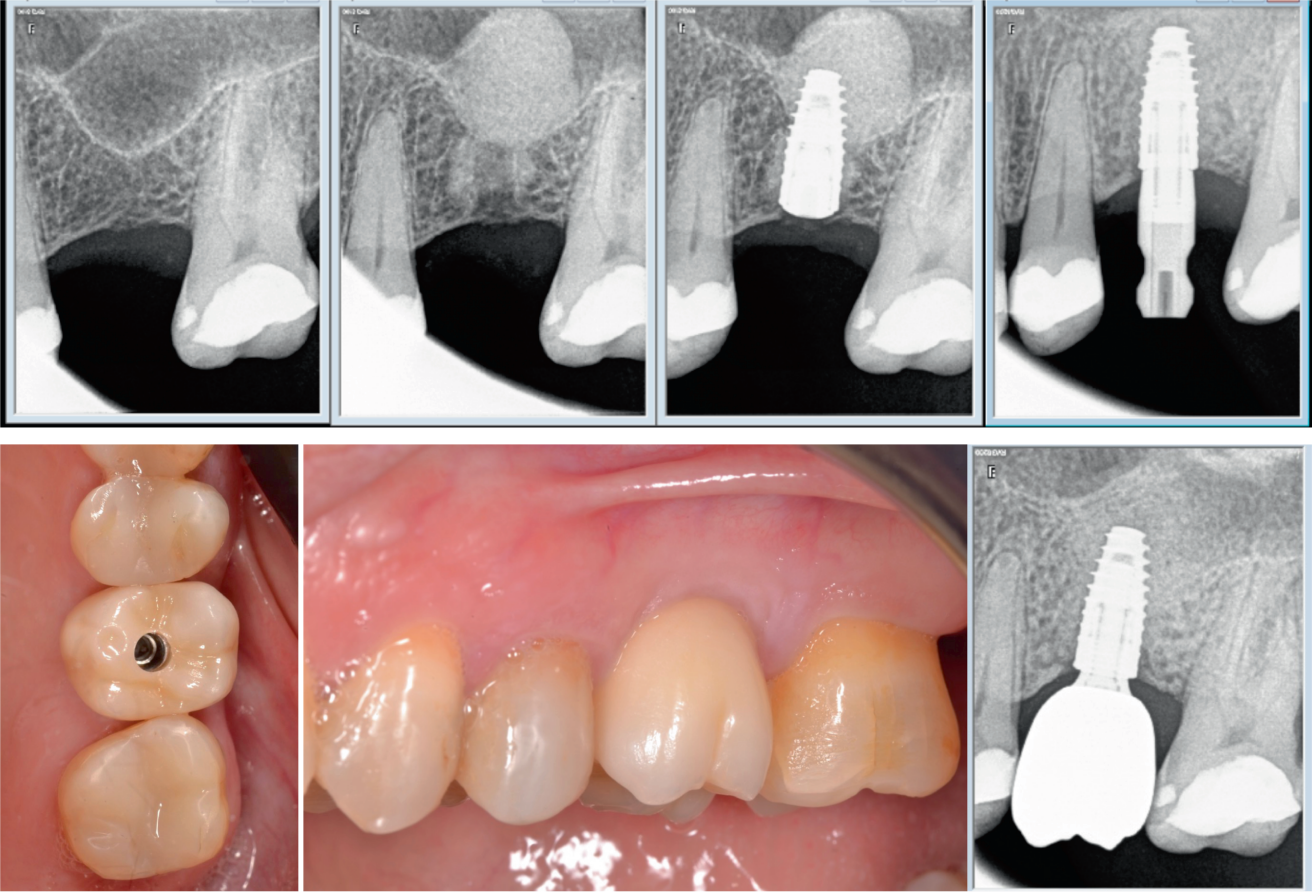
Sinus grafting with osseodensification—Protocol II (single site).

**FIGURE 5:**
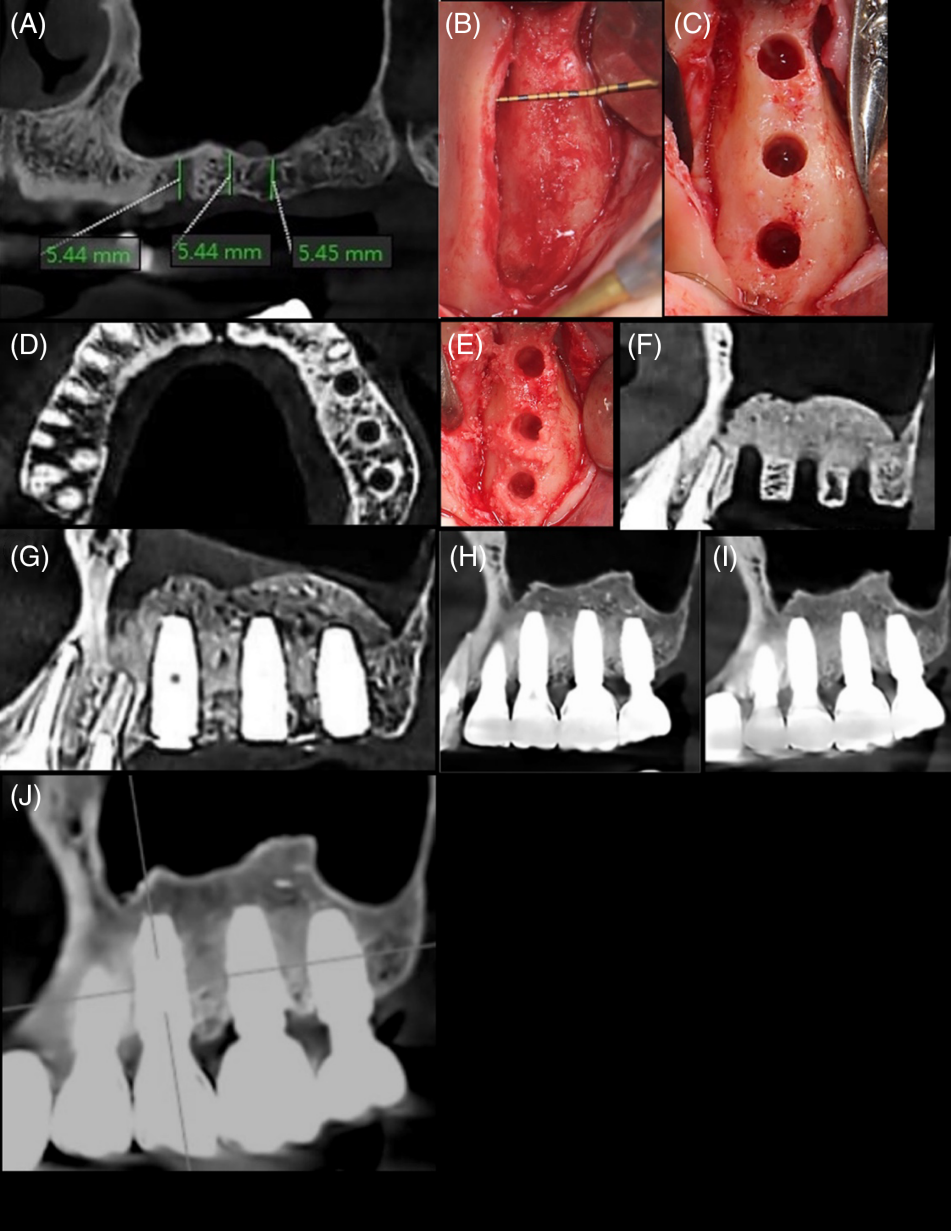
(A) OD Sinus Protocol II (maxillary left quadrant): CBCT scan depicting sinus pneumatization with 5.5 mm alveolar ridge height. (B) Occlusal clinical view of maxillary left quadrant with missing second premolar and upper first and second molar. Adequate alveolar ridge width >5 mm is evident. (C) Occlusal clinical view of three osteotomies preparation with osseodensification. (D) CBCT scan demonstrating the three sperate osteotomies with evident of osseodensification. (E) Occlusal clinical view of three osteotomies preparation with OD crestal sinus grafting protocol II utilizing Allograft. (F) CBCT radiograph demonstrating adequate lift with no evidence of membrane perforation and efficient grafting. (G) CBCT scan demonstrating adequate lift with grafting with no evidence of membrane perforation with simultaneous implants placement. (H–J) CBCT follow‐up of 1 year, 3 years, and 4 years, respectively, demonstrating a stable osseointegration of the three implants placed in the grafted upper left sinus with maintained crestal bone and sinus graft height in area. Clinical case is a courtesy of Dr. Chang WenPing.

Figures 4 and 5 are single unit and quadrant, respectively, case examples of clinical procedures for OD sinus lift Protocol II.

#### 4.2.5 Protocol III—minimum RBH 2–3 mm, minimum alveolar width needed = 7 mm

Cases of more severe posterior maxillary atrophy have also been the subject of clinical investigations, for instance, when the minimum RBH is between 2 and 3 mm and minimum alveolar width is of 7 mm. An RCT comparing the lateral window technique with OD drilling for SFE in cases of RBH ≤4 mm showed that success rates of implants were similar and that all implants were uneventfully restored. However, OD drilling significantly outperformed LW technique in pain experience, surgery duration, postoperative edema, and analgesic intake.^95^ A prospective clinical study of crestal SFE using OD drilling with simultaneous implant placement covered Protocols II and III (residual bone height ≥2 < 6 mm, mean 3.8 mm) showed high levels of primary and secondary implant stability and no sinus membrane perforation confirmed by cone‐beam CT scan postoperatively.^109^ A multicenter study evaluating the perforation rates of OD drilling covered all Protocols I, II, and III since the mean RBH was 5.1 mm (± 1.96 mm), ranging from 2 to 7 mm (256 sites had RBH between 3 and 5 mm, 249 sites had RBH >5 mm, and 165 sites had RBH ≤3 mm). Although the sinus membrane perforation rates were low (7.3%), regression analysis showed that severe atrophy (RBH ≤3 mm and between 3 and 5 mm) were identified as risk factors for membrane perforation. Tooth region (premolar and molar), implant site, healed, and fresh socket were not associated as risk factors for sinus membrane perforation.^117^

#### 4.2.6 Clinical suggested steps with OD sinus Protocol III

Step 1: measure bone height to the sinus floor.

Flap the soft tissue using instruments and technique normally used.

Step 2: OD bur (3.0), OD mode to sinus floor.

Avoid using a pilot drill, as well as densifying bur VT1525 (2.0). Depending upon the implant type and diameter selected for the site, begin with OD bur VT2535 (3.0). Change the surgical motor to reverse‐densifying mode (counterclockwise drill speed 800–1200 rpm with copious irrigation). Begin running the bur into the osteotomy until reaching the dense sinus floor. Confirm bur position with a radiograph.

Step 3: enter with OD bur (4.0) OD mode up to 3 mm past the sinus floor.

Use the next wider densifying bur VT3545 (4.0) and advance it into the previously created osteotomy with modulating pressure and a pumping motion. When feeling the haptic feedback of the bur reaching the dense sinus floor, modulate pressure with a pumping motion to advance past the sinus floor in 1 mm increments, up to 3 mm. Maximum bur advancement past the sinus floor, at any stage, must not exceed 3 mm. Bone will be pushed toward the apical end and will begin to gently lift the membrane and autograft compacted bone up to 3 mm. Confirm the bur vertical position with a radiograph.

Step 4: densifying bur VT4555 (5.0) OD mode up to 3 mm past the sinus floor. Use the sequential wider OD burs in densifying mode (counterclockwise drill speed 800–1200 rpm) with copious irrigation with pumping motion to achieve additional width with maximum membrane lift of 3 mm (in 1 mm increments) to reach final desired width for implant placement. OD burs must not advance more than 3 mm past the sinus floor at all times regardless of the OD bur diameter.

Step 5: propel allograft/bone substitute.

After achieving the final planned osteotomy diameter, fill the osteotomy with a well hydrated, mainly cancellous, allograft, or alloplast putty. Use the last OD bur used in step 4 in densifying mode (counterclockwise) with low speed 150–200 rpm with no irrigation to propel the allograft/ alloplast putty into the sinus. The OD bur must only facilitate the allograft material compaction to further lift the sinus membrane, and not to advance beyond the sinus floor more than 2–3 mm. Repeat the graft propelling step to facilitate additional membrane lift as needed according to implant length.

Step 6: place implant.

**FIGURE 6:**
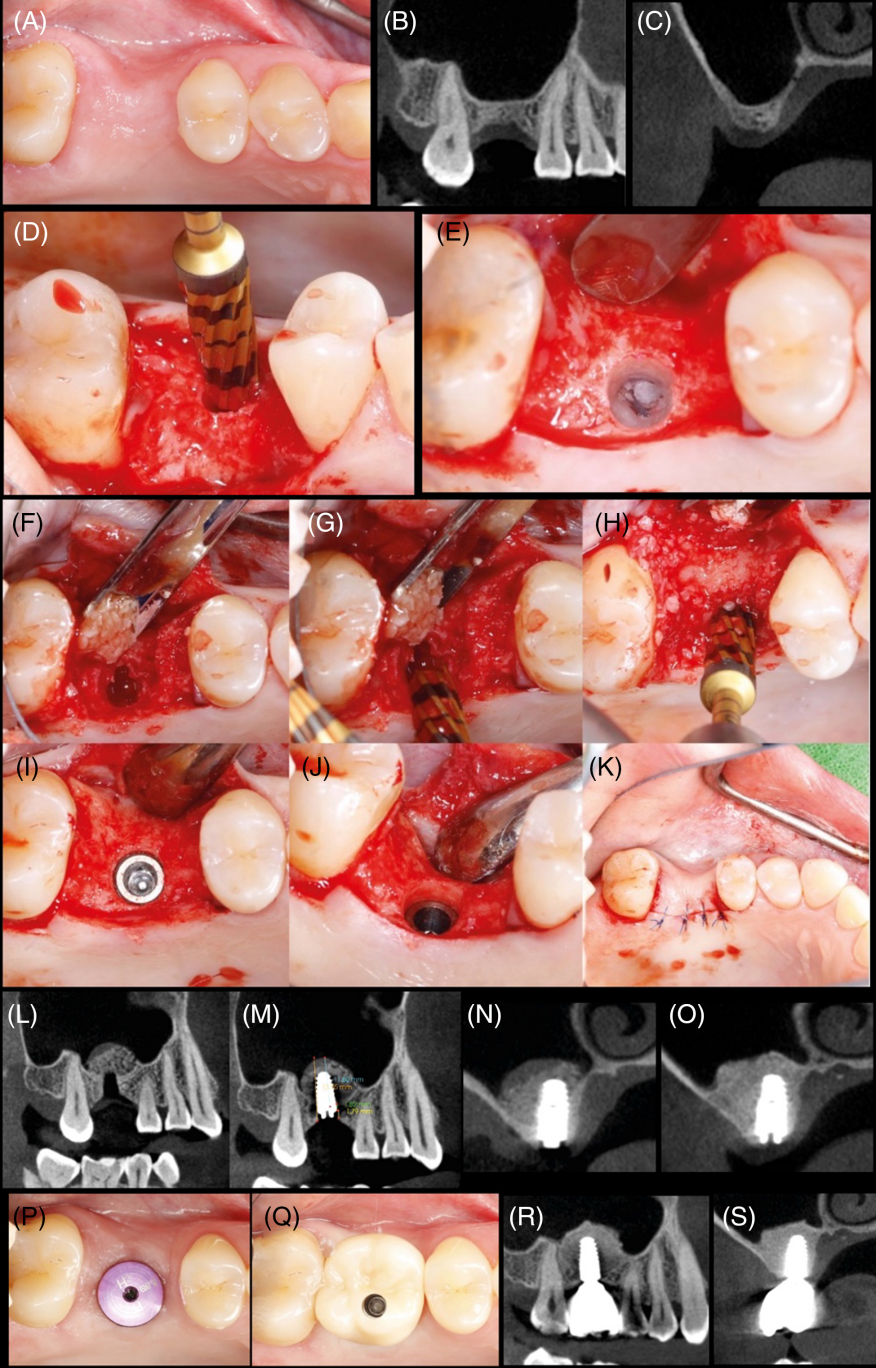
(A–S) Sinus grafting with osseodensification Protocol III—clinical example courtesy of Dr.Marcel Firlej. (A) Sinus grafting with osseodenfication Protocol III/maxillary right first molar site. (B,C) Radiographs of evident sinus pneumatization with crestal height deficiency and 3 mm alveolar ridge height. (D) Occlusal clinical view of osteotomy preparation in site with osseodensification. Utilizing OD bur VT3545 (4.0) in osseodensification mode (1000 RPM CCW with irrigation) to enter the sinus in the molar site to facilitate initial Schniederian membrane separation off the sinus bony bed with autogenous bone compaction grafting into the sinus. (E) Note site with intact Schneiderian membrane. (F–K) Occlusal clinical view of final implant osteotomy created with particulate allograft in the molar area grafting the sinus, utilizing OD bur at 100–200 RPM with no irrigation in CCW in the molar site grafting the sinus with allograft, implant placement, and suture. (L–N) CBCT verification day of sinus augmentation surgery with cancellous/cortical allograft. Note the extent of the Schneidarian membrane lift, the graft, and placed implant. (O) CBCT verification prior to implant uncover at 3 months post implant placement showing sinus graft with implant integration. (P) Occlusal clinical view of the site with healing abutment. (Q) Occlusal clinical view of restoration delivery and (R) Final CBCT. (S) 3‐years follow‐up, CBCT scan demonstrating a stable osseointegration of the implant placed in maxillary right first molar region with maintained crestal bone and sinus graft height.

**FIGURE 7:**
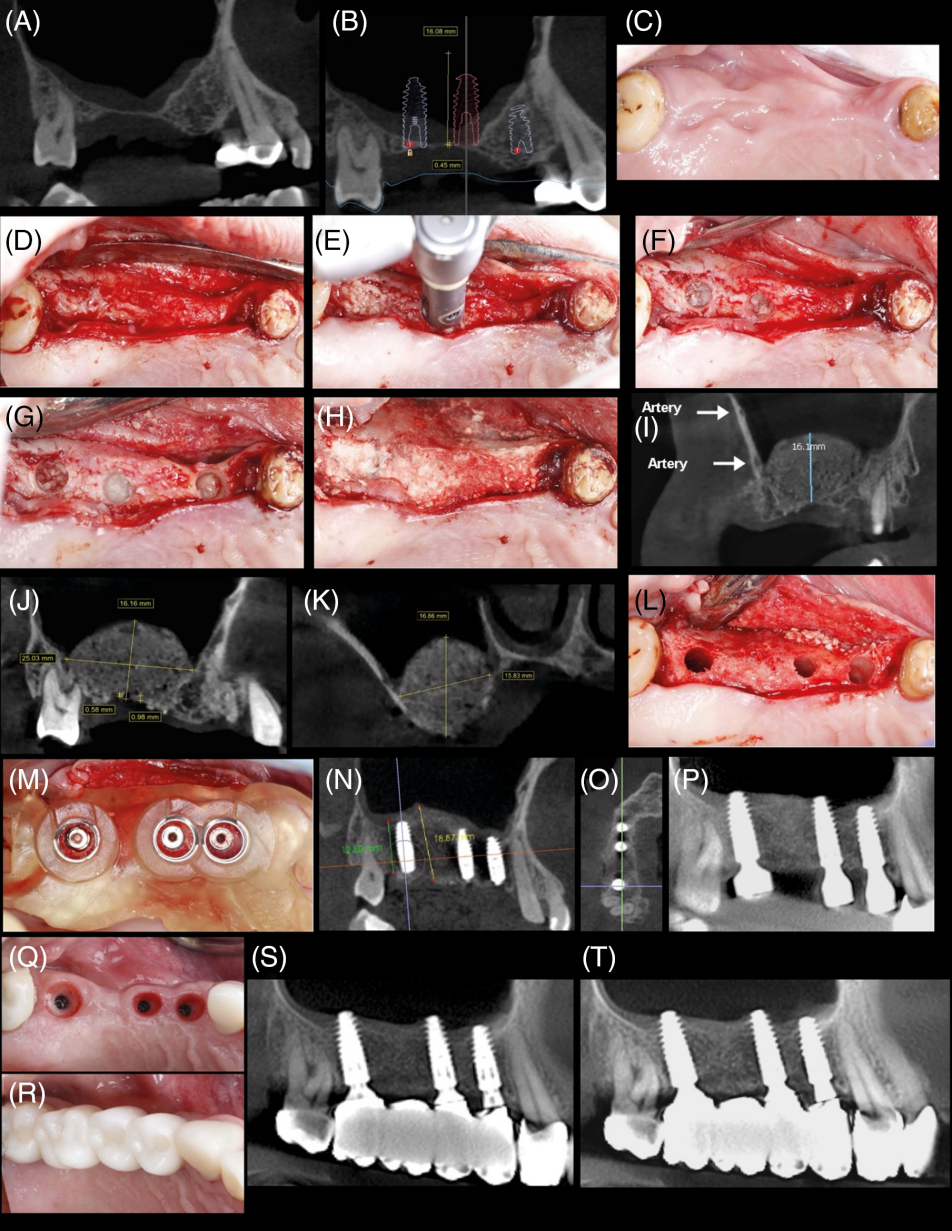
(A–T) Advanced sinus grafting with osseodensification 2‐stage Protocol III in right maxillary quadrant (courtesy Dr. Marcel Firlej). Combined crestal sinus grafting with localized lateral ridge expansion of severally resorbed alveolar ridge with second‐stage approach post augmentation for implants placement: patient presented with missing maxillary right first and second premolar, and first and second molar with severe crestal alveolar bone height deficiency and localized horizontal alveolar ridge resorption in the right first premolar site. Crestal bone height ranged from <1 mm in molars sites to ≥5 mm in premolar area. (A) Initial CBCT scan of the maxillary right quadrant; (B) digital treatment plan; (C) initial clinical occlusal view of the maxillary right edentulous area. (D) Occlusal clinical view with flap reflection showing localized narrow alveolar ridge in the first and second premolar sites. (E,F) OD bur VT4555 (5.0) was used in osseodensification mode (1000 RPM CCW with irrigation) with vertical G‐stop to enter the sinus in molars sites to facilitate initial Schneiderian membrane separation off the sinus bony bed with autogenous bone compaction grafting into the sinus. Note molar sites osteotomies with intact Schneiderian membrane. (G) Occlusal clinical view of the final osteotomy created with particulate allograft in the molar area grafting the sinus, allograft was slowly compacted with OD bur VT4555 (5.0) running in 100 RPM to achieve adequate sinus grafting. Ridge expansion was done in the premolar site using OD burs according to osseodensification ridge expansion protocol. (H) Occlusal clinical view of the final three osteotomies fully grafted with cancellous/cortical allograft. (I) CBCT scans for verification in the day of sinus augmentation surgery with cancellous/cortical allograft. Note the extent of the membrane lift to 16 mm. (J) 4 months post‐surgery follow‐up CBCT confirming adequate healing of the augmented upper right sinus and (K) is after 8 months post‐surgery and prior to implant placement. (L,M) Occlusal clinical view of implants osteotomies and placement in area of first premolar, first molar, and second molar. Densifying burs were used in osseodensification mode (1000 RPM CCW) to create the osteotomies through the surgical C‐guide (Versah, LLC). (N,O) CBCT scans of the implant's placements (8 months post initial sinus grafting / ridge expansion). Note: adequate vertical and horizonal upper right alveolar ridge augmentation with sinus grafting. (P) CBCT scan at day of implant uncover at 4 months post implant placement. (Q,R) Occlusal views and (S) CBCT scan of healing at 6‐months post implant placement with implants supporting a fixed dental prosthesis. (T) 3‐years follow‐up of CBCT scan demonstrating a stable osseointegration of the three implants placed in the grafted sinus with maintained crestal bone and sinus graft height.

Place the implant into the osteotomy. If using the surgical motor to tap the implant into place, the unit may stop when reaching the placement torque maximum. Finish placing the implant to depth with a torque indicating wrench. Figures 6 and 7 are examples single unit and quadrant cases, respectively, of clinical procedures for sinus lift with OD burs in protocol III.

#### 4.2.7 Concluding remarks

Implant treatment success or failure is determined by several factors including patient health status, surgeon skill, implant design, osteotomy site preparation, and design of the prosthetic reconstruction. OD burs have been shown as a safe alternative with high predictability for transcrestal SFE, even though severe posterior maxillary atrophy (RBH ≤3 mm) presented as a risk factor for increased membrane perforation rate. However, it is important to note that the studies included in this review exclusively used the patented and first system launched in the market since evidence has been made available in peer reviewed health science databases for this technique. Extrapolation of the information presented herein to other manufacturers' osseodensification drilling systems should be made with caution.

## AUTHOR CONTRIBUTIONS

**João Gaspar**: Conceptualization; draft writing; clinical cases presented; review and editing; proofreading the final version. **Ziv Mazor**: Writing; review and editing; proofreading the final version. **Estevam Bonfante**: Conceptualization; draft writing; review and editing; proofreading the final version.

## CONFLICT OF INTEREST STATEMENT

The authors declare no conflicts of interest.

## Data Availability

Data sharing is not applicable to this article as no new data were created or analyzed in this study.
